# Measured Prenatal and Estimated Postnatal Levels of Polychlorinated Biphenyls (PCBs) and ADHD-Related Behaviors in 8-Year-Old Children

**DOI:** 10.1289/ehp.1408084

**Published:** 2015-03-13

**Authors:** Marc-André Verner, Jaime E. Hart, Sharon K. Sagiv, David C. Bellinger, Larisa M. Altshul, Susan A. Korrick

**Affiliations:** 1 Channing Division of Network Medicine, Brigham and Women’s Hospital, Department of Medicine, Harvard Medical School, Boston, Massachusetts, USA; 2 Institute of Environmental Medicine, Karolinska Institutet, Stockholm, Sweden; 3 Department of Environmental Health, Harvard School of Public Health, Boston, Massachusetts, USA; 4 Department of Environmental Health, Boston University School of Public Health, Boston, Massachusetts, USA; 5 Children’s Hospital, Harvard Medical School, Boston, Massachusetts, USA; 6 Environmental Health and Engineering Inc., Needham, Massachusetts, USA

## Abstract

**Background:**

Epidemiologic studies of postnatal PCB exposure and behavior have not reported consistent evidence of adverse associations, possibly because of challenges in exposure estimation. We previously developed a pharmacokinetic model to improve estimation of children’s PCB exposure.

**Objectives:**

We aimed to assess whether estimated serum PCB levels in infancy are associated with attention deficit/hyperactivity disorder (ADHD)–related behaviors at 8 years of age among children whose cord serum PCB levels were previously shown to be associated with ADHD-related behaviors.

**Methods:**

We used a pharmacokinetic model to estimate monthly serum polychlorinated biphenyl (PCB)–153 levels in 441 infants (ages 1–12 months) based on parameters such as breastfeeding and cord serum PCB-153 levels. Behavior was evaluated at age 8 using the Conners’ Rating Scale for Teachers (CRS-T). Associations between PCB-153 levels and ADHD-related CRS-T indices were assessed using multivariable quantile regression at the 50th and 75th percentiles of CRS-T scores, where higher percentiles reflect more adverse behaviors.

**Results:**

Cord serum PCB-153 levels (median, 38 ng/g lipids) were associated with ADHD-related behaviors, although statistical significance was observed with quantile regression models only at the 75th percentile. Associations with postnatal exposure estimates were attenuated. For example, hyperactive-impulsive behavior scores at age 8 years were 0.9 points (95% CI: 0.2, 2.5), 0.5 points (95% CI: 0.3, 2.3), and 0.3 points (95% CI: –0.2, 1.5) higher in association with interquartile range increases in serum PCB-153 at birth, 2 months, and 12 months of age, respectively.

**Conclusions:**

Associations between estimated postnatal PCB-153 exposures and ADHD-related behaviors at 8 years of age were weaker than associations with PCB-153 concentrations measured in cord serum at birth.

**Citation:**

Verner MA, Hart JE, Sagiv SK, Bellinger DC, Altshul LM, Korrick SA. 2015. Measured prenatal and estimated postnatal levels of polychlorinated biphenyls (PCBs) and ADHD-related behaviors in 8-year-old children. Environ Health Perspect 123:888–894; http://dx.doi.org/10.1289/ehp.1408084

## Introduction

Evidence from experimental and human studies (reviewed by Eubig et al. 2010) suggests that prenatal exposure to polychlorinated biphenyls (PCBs) can adversely affect children’s behavior. During infancy, breastfeeding represents the primary, if not exclusive, source of postnatal PCB exposure and can lead to childhood blood PCB levels that exceed prenatal levels as measured in cord blood (Ayotte et al. 2003). However, the few epidemiologic studies that have assessed the association between postnatal exposure to PCBs and attention and activity, for example, have not reported consistent evidence of adverse effects (Jacobson and Jacobson 2003; Jacobson et al. 1990; Plusquellec et al. 2010; Verner et al. 2010; Vreugdenhil et al. 2002). This is surprising given the extensive brain development that occurs postnatally, including cell differentiation that has been shown to be altered by PCBs in human neuroprogenitor cells *in vitro* (Fritsche et al. 2005).

Failure to observe associations between postnatal PCB exposure and neurobehavior may be related to methodological and logistical challenges to adequately characterizing exposure during infancy and early childhood. Because PCB levels in breast milk correlate with prenatal levels as indicated by maternal and cord blood levels (Muckle et al. 2001), it is difficult to identify the independent role of lactational exposures on health measures. In addition, the association between lactational exposures and behavior is confounded by breastfeeding duration, which is positively associated with both infants’ blood PCB levels (Ayotte et al. 2003) and improved child neurobehavior (Julvez et al. 2007). Furthermore, estimating PCB body burden in infancy and early childhood presents additional obstacles. Because collecting serial blood samples in infancy is often impractical due to cost and logistical and ethical considerations, researchers have used metrics based on breast milk PCB levels and duration of breastfeeding (Jacobson et al. 1990; Vreugdenhil et al. 2002) or single blood measurements in later childhood (Jacobson and Jacobson 2003; Jacobson et al. 1990). None of these approaches allow estimation of PCB exposure at different periods of postnatal development, some of which may be more sensitive to PCBs than others.

As an efficient alternative, we recently developed a pharmacokinetic model based on maternal and child physiology and biochemistry to estimate profiles of persistent organic pollutant levels in children’s body lipids (including blood lipids) during and after breastfeeding (Verner et al. 2013). The predictive value of this model was subsequently evaluated using data from Inuit and Slovak birth cohorts in which children had their blood sampled at around 6 months of age (Verner et al. 2013). Simulations based on cord blood PCB-153 levels and information on maternal prepregnancy body weight and child gestational age, birth weight, sex, infant weight, and exclusive and total breastfeeding duration explained 74% and 57% of the variability in blood PCB-153 levels of infants enrolled in the Inuit and Slovak cohorts, respectively. Using a pharmacokinetic model to estimate month-by-month postnatal blood PCB-153 levels through age 11 months in Inuit children, we observed an association between measured cord blood PCB-153 levels and inattention and between estimated postnatal blood PCB-153 levels (especially around the 4th month) and activity at age 11 months (Verner et al. 2010). This finding, together with the fact that different neurodevelopmental processes occur in different regions of the brain during distinct prenatal and postnatal periods (Rice and Barone 2000), supports the potential for differential PCB effects on behavior depending on exposure timing. Whether the relation of early-life PCB exposures with behavior in later childhood varies with prenatal versus infancy exposure timing has yet to be studied. Identifying developmental periods of differential PCB susceptibility can be important to public health strategies for exposure mitigation.

In a longitudinal birth cohort of mothers and children living near a PCB-contaminated site in New Bedford, Massachusetts (USA) (Choi et al. 2006), cord serum PCB levels were associated with an increased risk of attention deficit/hyperactivity disorder (ADHD)–related behaviors at 8 years of age measured using the Conners’ Rating Scale for Teachers (CRS-T), a standardized questionnaire for estimating the frequency of adverse behaviors in children (Sagiv et al. 2010). In the same cohort, we aimed to evaluate the differential effects of prenatal and postnatal exposures on children’s behavior by *a*) estimating children’s monthly serum PCB-153 levels during the first year of life using the above-described validated pharmacokinetic model, and *b*) evaluating the association of measured cord serum and estimated monthly infant serum PCB-153 levels with behavior using quantile regression at the 50th and 75th percentiles of CRS-T scores. This statistical approach allowed us to evaluate whether PCB exposures are more strongly associated with ADHD-related behaviors in children with more adverse behaviors at 8 years (75th percentile of CRS-T scores), who may represent a more susceptible population because of genetic factors or other early environmental exposures.

## Methods

*Study population*. Mothers residing in towns adjacent to the PCB-contaminated New Bedford Harbor were invited to participate in the New Bedford Cohort at the time of delivery if they were > 18 years of age, gave birth via vaginal delivery, and spoke English or Portuguese (Korrick et al. 2000). A total of 788 newborns were enrolled between 1993 and 1998. We restricted our analyses to singleton births (*n* = 782) with cord serum PCB measures (*n* = 746) and information on duration of exclusive and total breastfeeding (*n* = 581), and to those who participated in 8-year follow up assessments with complete CRS-T evaluations (*n* = 567). A total of 441 of the 567 children had complete covariate data and were included in our analyses. This study was approved by the human subjects committees of Harvard School of Public Health and Brigham and Women’s Hospital, Boston, Massachusetts and Southcoast Hospitals Group, New Bedford, Massachusetts. Mothers gave written informed consent before examination.

*Laboratory measurement methods*. Cord blood sampling, analytical procedures, and PCB levels have been described elsewhere (Korrick et al. 2000). As part of previous research on prenatal PCB exposures in the New Bedford Cohort, cord blood was collected at birth, and cord serum levels of 51 PCB congeners were measured at the Harvard School of Public Health Organic Chemistry Laboratory (Boston, MA). Serum samples underwent liquid–liquid extraction followed by analysis by gas chromatography with electron capture detection. Because of limited sample volume, serum lipids were not measured. The concentration of lipids in cord serum was measured in 12 randomly selected, discarded anonymous samples from the study hospital where New Bedford Cohort infants were born (mean, 1.7 g lipid/L serum). Because the pharmacokinetic model uses PCB levels in lipids, we expressed cord serum PCB levels on a lipid basis using the same 1.7-g/L lipid concentration for all cord serum samples.

**Figure 1:**
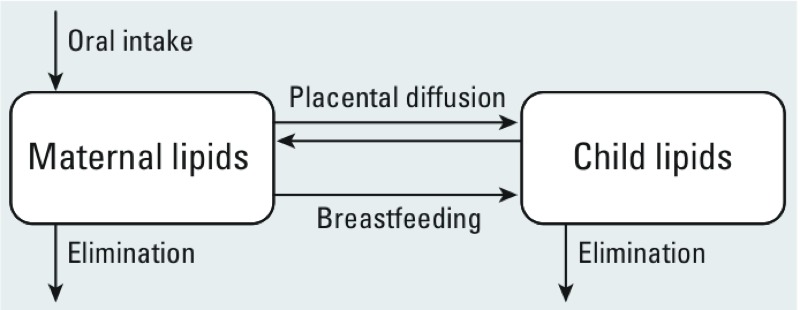
Conceptual representation of the pharmacokinetic model (Verner et al. 2013). Reproduced with permission from *Environmental Health Perspectives*.

*Postnatal exposure assessment*. We used PCB-153 as a proxy for total PCB exposure because of its long half-life in humans (Ritter et al. 2011) and its correlation with other prevalent PCB congeners in this population (Spearman correlation coefficients of 0.72 for PCB-118, 0.81 for PCB-138, and 0.73 for PCB-180). We estimated children’s serum lipid PCB-153 levels at each of the first 12 postnatal months using our previously published pharmacokinetic model (Verner et al. 2013). This model simulates lifetime exposure in the mother, transplacental diffusion during pregnancy, and transfer via breastfeeding after delivery (Figure 1). Both the mother and the child are represented in the model, and only body lipids are included because PCB-153 is highly lipophilic and binds almost exclusively to lipids. The model assumes a homogeneous distribution across body lipids, including serum lipids. Maternal exposure was parameterized as a constant daily dose (nanograms per kilogram body weight) calibrated to match measured cord serum PCB-153 levels at birth. Infants’ postnatal exposure through age 12 months was attributed entirely to breastfeeding.

The pharmacokinetic model runs on subject-specific information to generate individualized profiles of serum PCB-153 levels during infancy. Here, we used the New Bedford Cohort participants’ information on maternal age at delivery, prepregnancy body weight, and gestational weight gain; and child duration of exclusive and total breastfeeding, gestational age at birth, birth weight, and weight at the time of the 8-year behavioral assessment (birth weight and weight at 8 years were used to estimate each infant’s growth curve between birth and 12 months). Data on duration of breastfeeding was collected as categories: no breastfeeding, < 1 month, 1–3 months, 4–6 months, 7–9 months, and > 9 months. Because the pharmacokinetic model uses continuous data, these categories were transformed to breastfeeding durations of 0, 0.5, 2, 5, 8, and 10 months, respectively. Because subject-specific information was unavailable for certain model inputs, we used the same assumptions for all individuals. Between the end of exclusive breastfeeding (when formula or solid food was introduced) and the end of total breastfeeding, we assumed breast milk consumption to be 50% of intake during exclusive breastfeeding. To model maternal weight changes postpartum, we assumed body weight to be 2 kg higher than prepregnancy body weight 6 months after delivery (Butte et al. 2003) and to return to prepregnancy body weight 12 months after delivery. The same assumptions were used in the model development and validation study (Verner et al. 2013).

*Behavioral assessment*. The CRS-T is a 59-item standardized questionnaire used to assess the frequency of a wide range of problem behaviors in children as reported by their teachers (Conners 1997). It has been used in research among nonclinical populations but is also used in the diagnosis and treatment of children with behavioral problems, including problems with attention, hyperactivity, and impulse control that are associated with ADHD. The CRS-T has been shown to be both sensitive and specific in differentiating children with clinically diagnosed ADHD from children with neurotypical behavior (Tripp et al. 2006). Teachers of children participating in the study’s 8-year follow-up were asked to complete the CRS-T questionnaire. The four CRS-T subscales pertaining to ADHD-related behaviors were used in this analysis: *a*) Conners’ ADHD index, *b*) *Diagnostic and Statistical Manual of Mental Disorders,* Fourth Edition (DSM-IV) Inattentive index (American Psychiatric Association 1994), *c*) DSM-IV Hyperactive-Impulsive index, and *d*) DSM-IV Total index (inattentive and hyperactive-impulsive behaviors combined) (Conners 1997). CRS-T scores are standardized to age- and sex-adjusted *T*-scores with a mean (± SD) of 50 ± 10 where more frequent adverse behaviors are characterized by higher CRS-T scores.

*Covariate assessment*. Information about mother–child sociodemographics, medical history, weight, lifestyle, and dietary habits, including infant feeding, were collected via study questionnaires administered during study evaluations of each child at approximately 2 weeks after birth and at ages 6 months (for a subset of the cohort) and 8 years. Additional medical history, weight, pregnancy history, and birth outcome data were collected by review of the study hospital’s labor and delivery medical records as well as outpatient pediatric medical records with data abstracted between birth and age 8 years. In addition, child weight at 8 years was measured as part of the 8-year study evaluation.

*Statistical analysis*. We used multivariable quantile regression, a method that has not been used in prior analyses of behavior in this cohort, to evaluate the associations of measured (cord serum) and simulated (postnatal) serum lipid PCB-153 levels with children’s behavior. Quantile regression differs from ordinary least square (OLS) linear regression in that it minimizes the sum of absolute residuals rather than the squared residuals, thus reducing the influence of outliers and making it more robust to departure from ordinary least square model assumptions (Koenker and Bassett 1978). Quantile regression’s robustness to non-Gaussian distributions made it an appropriate regression method for the skewed CRS-T scores. In addition, quantile regression allows for assessment of associations at any quantile of the dependent variable by symmetrically (median) or asymmetrically (other quantiles) weighting positive and negative residuals. For example, in quantile regression at the 75th percentile, underpredictions (positive residuals) are given a weight of 0.75 whereas overpredictions (negative residuals) are given a weight of 0.25. This model would indicate the change in the 75th percentile of the outcome value associated with each unit change in a predictor variable. Quantile regression uses all available data regardless of the quantile of interest, thereby providing overall estimates of association. Evaluating associations at different percentiles is particularly useful when exposure is hypothesized to affect certain percentiles of the dependent variable to a greater extent than the mean or median (Beyerlein 2014). For example, children with poorer neurocognitive skills may be particularly susceptible to neurotoxicant effects, as observed in a study of lead and cognitive function by Miranda et al. (2009). In our study, one could hypothesize that children who are predisposed to ADHD-related behaviors due to genetic and early-life environmental factors—those most likely to be in the higher percentiles of CRS-T scores at age 8 years—may be more sensitive to early exposures to PCBs. We ran quantile regressions for the study at the 50th percentile (roughly analogous to OLS linear regression at the mean) and at the 75th percentile (more adverse behaviors) of CRS-T scores. We picked the 75th percentile *a priori* to optimize our assessment of PCB effects on children with worse scores without compromising the precision of the analyses. Because the distribution in estimated children’s PCB-153 levels changes during development, and because regression coefficients are influenced by the mean and SD of exposure estimates, we expressed the change in CRS-T scores by the interquartile range (IQR) increase in PCB-153 levels for each time period to make coefficients comparable across exposure periods.

We included the following potential confounders (related to exposures and outcomes) or control variables (related to outcomes) in regression models based on *a priori* considerations: maternal prepregnancy weight, gestational weight gain categories (inadequate, adequate, excessive) using the Institute of Medicine recommendations (Rasmussen and Yaktine 2009), characteristics at delivery (age, marital status, education, parity), seafood consumption during pregnancy, use of tobacco and alcohol during pregnancy and use of illicit drugs in the year before delivery, and IQ at 8-year follow-up; total household income (at delivery) and Home Observation for Measurement of the Environment (HOME) score (Caldwell and Bradley 1984) at 8-year assessment; and child sex, race, cord blood lead level, ADHD medication use, school type, and age at the CRS-T evaluation. Because simulated postnatal serum PCB-153 levels are strongly correlated with cord serum PCB-153 levels and duration of breastfeeding, we did not adjust for these two measures in our final regression models; however, we performed secondary sensitivity analyses adjusting for these factors. We also did secondary quantile regression analyses restricted to the subset of children who were ever breastfed.

## Results

Table 1 provides a summary of study participants’ sociodemographic, lifestyle, and exposure characteristics. At the time of delivery, mothers in this study were, on average, 27 years of age, 38% were unmarried, and about one-third lived in low-income households with annual incomes < $20,000. Only 31% of the women had a gestational weight gain within the recommendations of the Institute of Medicine (Rasmussen and Yaktine 2009), whereas 51% gained more weight. Children were on average 8 years of age at behavioral evaluation, were predominantly white (69%), and only 54% were ever breastfed. Complete covariate data were available for 441 of the 567 children identified for study. Of all children enrolled in the New Bedford Cohort, those in this analysis (*n* = 441) were generally more sociodemographically advantaged than those excluded (*n* = 347) (data not shown). For example, included mothers were older, had more education, were more likely to be married, and had a higher IQ; their children were younger at CRS-T examination and more likely to be breastfed than those excluded. Conversely, included mothers smoked more during pregnancy and their children had higher cord blood lead levels than those excluded (data not shown). A substantial proportion (29%) of mothers excluded from the analyses had missing prenatal smoking information, which may, in part, account for the apparent increased smoking among included participants.

**Table 1:** Sociodemographic, lifestyle, and exposure characteristics of New Bedford Cohort mothers and children born 1993–1998 included in the analyses (*n *= 441).

Characteristic	*n* (%)	Mean ± SD	Range
Maternal characteristics
Age at delivery (years)		27.2 ± 5.3	18–40
Prepregnancy BMI (kg/m^2^)
< 25	329 (75)
≥ 25	112 (25)
Gestational weight gain
< IOM^*a*^ recommendations	79 (18)
Within IOM recommendations	135 (31)
> IOM recommendations	227 (51)
Maternal education at delivery
≤ High school	239 (54)
College or university	202 (46)
Annual household income at delivery
< $20,000	153 (35)
$20,000–$40,000	142 (32)
> $40,000	146 (33)
Parity before index child		0.9 ± 0.9	0–4
Marital status at delivery
Married	272 (62)
Never married/separated/divorced	169 (38)
Intellectual quotient at 8-year assessment		98.8 ± 10.4	57–121
Average number of cigarettes per day during pregnancy		2.9 ± 5.9	0–40
Alcohol consumption during pregnancy
< 1 serving/month	412 (93)
≥ 1 serving/month	29 (7)
Used illicit drugs in the year before delivery?
No	380 (86)
Yes	61 (14)
Fish and seafood consumption during pregnancy
≤ 2 servings/week	214 (49)
> 2 servings/week	227 (51)
Child characteristics
Sex
Male	222 (50)
Female	219 (50)
Race/ethnicity
White	304 (69)
Other	137 (31)
Age at evaluation (years)		8.1 ± 0.5	7–11
HOME score at 8-year assessment		45.7 ± 5.3	28–57
School type
Public	399 (90)
Private	42 (10)
Breastfeeding
Never	202 (46)
< 1 month	61 (14)
1–3 months	52 (12)
4–6 months	46 (10)
7–9 months	32 (7)
> 9 months	48 (11)
ADHD medication
Yes	30 (7)
No	411 (93)
Contaminant levels
Cord blood PCB-153 (ng/g lipids)		53.7 ± 67.1	0–790.6
Cord blood lead (μg/dL)		1.4 ± 1.0	0–9.4
BMI, body mass index. ^***a***^The Institute of Medicine (Rasmussen and Yaktine 2009) recommends gestational weight gains of 12.7–18.1 kg in underweight women (prepregnancy BMI < 18.5), 11.3–15.9 kg in normal-weight women (prepregnancy BMI 18.5–24.9), 6.8–11.3 kg in overweight women (prepregnancy BMI 25.0–29.9), and 5.0–9.1 kg in obese women (prepregnancy BMI > 30).			

**Figure 2:**
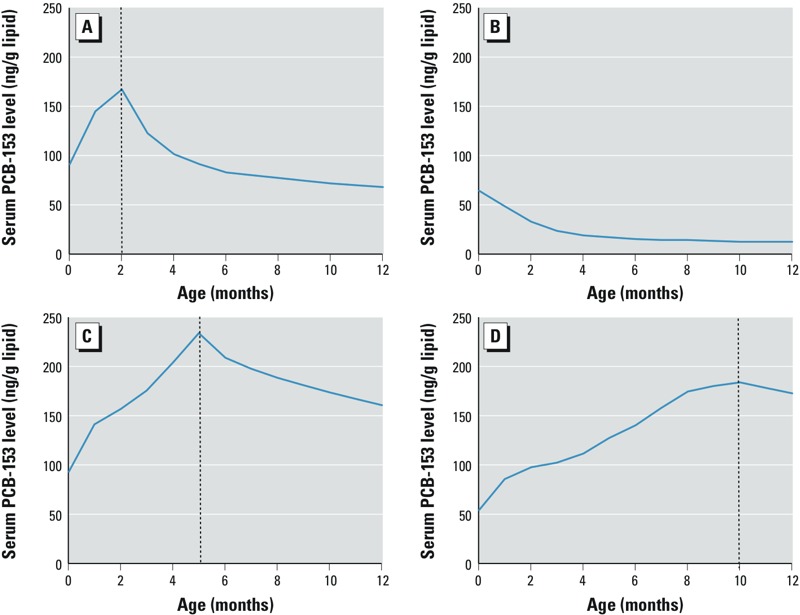
Examples of measured cord serum lipid and estimated postnatal serum lipid PCB-153 levels over the first 12 months of life in four study infants with different durations of breastfeeding and cord serum levels: (*A*) 2 months of breastfeeding, 91 ng PCB-153/g lipids in cord serum; (*B*) no breastfeeding, 64 ng PCB-153/g lipids in cord serum; (*C*) 5 months of breastfeeding, 93 ng PCB-153/g lipids in cord serum; (*D*) 10 months of breastfeeding, 54 ng PCB-153/g lipids in cord serum. The vertical dotted lines represent the age at which breastfeeding stopped for the three infants who were breastfed.

We simulated PCB-153 levels in infants using a pharmacokinetic model that assumes that all postnatal exposure during the first year of life is from breastfeeding. Four representative plots of PCB-153 levels are shown for infants with different durations of breastfeeding in Figure 2. Estimated infants’ serum PCB-153 increased during lactation, peaked at the end of breastfeeding, and subsequently decreased with cessation of postnatal exposure and dilution of body burden with growth and expanding body lipids. Among breastfed infants, the increase in estimated serum PCB-153 levels over time was steeper in infants whose measured cord serum level at birth was higher (Figure 2A and C compared with D). Levels of serum PCB-153 that were accumulated prenatally declined rapidly after delivery in infants who were not exposed via breastfeeding (Figure 2B). Because of the relatively low prevalence of breastfeeding in this population, median estimated serum PCB-153 levels declined from 38 ng/g lipids at birth to 30 ng/g lipids at 2 months and 14 ng/g lipids at 12 months (see Supplemental Material, Figure S1) despite the fact that levels increased in breastfed infants. Estimated PCB-153 levels in infants’ serum were highly correlated with measured cord serum levels. The Spearman correlation coefficients between cord serum PCB-153 levels and postnatal estimates decreased from the first month (*r* = 0.93) to the 6th month (*r* = 0.70) and stabilized around 0.67 thereafter. On the other hand, the Spearman correlation between the total duration of breastfeeding and serum PCB-153 levels increased from 0.06 at birth to 0.68 at 6 months of age and stabilized around 0.70 thereafter (see Supplemental Material, Figure S2). This increasing correlation was expected because the duration of breastfeeding is the most influential model parameter during the first year (Verner et al. 2013).

**Figure 3:**
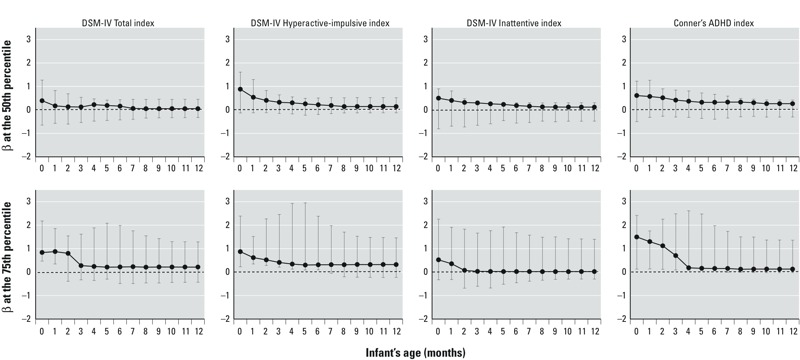
Quantile regression effect estimates for the 50th (top) and 75th percentiles (bottom) of scores for the Conners’ Rating Scale for Teachers (CRS-T) ADHD-related indices (*n *= 441). Circles represent the change in CRS-T score at age 8 years for an IQR increase in serum lipid PCB-153 levels measured in cord serum at birth and estimated for each month of infancy [time (months) and IQRs (ng/g lipids): 0 (birth) = 42; 1 = 49; 2 = 49; 3 = 44; 4 = 44; 5 = 45; 6 = 42; 7 = 42; 8 = 40; 9 = 39; 10 = 37; 11 = 36; 12 = 34]. Error bars represent the 95% CIs. Models were adjusted for maternal prepregnancy weight, gestational weight gain, characteristics at delivery (age, marital status, education, parity), seafood consumption during pregnancy, use of tobacco and alcohol during pregnancy and use of illicit drugs in the year before delivery, and IQ at 8-year follow-up; total household income (at delivery) and HOME score at 8-year assessment; and child sex, race, cord blood lead level, ADHD medication use, school type, and age at CRS-T evaluation.

In line with a previous study of the same cohort where children in the highest quartile of cord serum PCB levels had increased risks of ADHD-related behavior in a log risk regression analysis (Sagiv et al. 2010), results from the current quantile regression analyses also suggest that cord serum PCB-153 levels are associated with greater ADHD-related behavior as reflected in three of the four CRS-T subscales assessed (DSM-IV Total index, DSM-IV Hyperactive-Impulsive index, and Conners’ ADHD index). However, these associations were statistically significant in quantile regression models only at the 75th percentile (Figure 3). The association between PCB-153 and ADHD-related behaviors at age 8 years was stronger with measured cord serum levels than with estimated postnatal serum levels; the strength and significance of the associations attenuated substantially over time within the first few months of estimated postnatal exposure (Figure 3). The most sustained association with postnatal exposure estimates was a modest statistically significant association of estimated serum PCB-153 levels through age 6 months with greater DSM-IV Hyperactive-Impulsive index and Conners’ ADHD index behaviors at age 8 years (quantile regression models at the 75th percentile), but these associations also attenuated over time (Figure 3). For example, the increase in Hyperactive-Impulsive index behaviors at age 8 years associated with an IQR increase in PCB-153 levels was 0.9 point at birth [95% confidence interval (CI): 0.2, 2.5], 0.5 point at 2 months (95% CI: 0.3, 2.3), and 0.3 point at 12 months (95% CI: –0.2, 1.5). Because quantile regression does not assume any particular distribution of residuals, it is possible for CIs to be asymmetrical, which is the case in most of our regression models.

In sensitivity analyses, results were similar after models were adjusted for the total duration of breastfeeding (see Supplemental Material, Figure S3A). On the other hand, the modest associations with postnatal exposure disappeared when we adjusted for measured cord serum PCB-153 levels (see Supplemental Material, Figure S3B). Restricting analyses to breastfed children (see Supplemental Material, Figure S3C) did not affect regression coefficients substantially, though, consistent with the smaller number (*n* = 239), confidence intervals were wider than in main analyses. Results did not change materially when regression coefficients were expressed using the same IQR for all exposure periods (data not shown).

## Discussion

Results from animal (Holene et al. 1998; Rice 2000) and *in vitro* studies (Fritsche et al. 2005), and the relatively high PCB exposures that occur consequent to breastfeeding (Ayotte et al. 2003; Lanting et al. 1998; Patandin et al. 1997) support likely adverse neurobehavioral impacts of PCB exposure in infancy, including adverse impacts on impulse control (Verner et al. 2010). We used a pharmacokinetic model to estimate monthly serum PCB-153 levels in the first year of life and to assess the relation of these exposure estimates with ADHD-related behavior at age 8 years, including hyperactivity and impulsivity, in the New Bedford Cohort.

Cord serum PCB-153 levels were significantly associated with the DSM-IV Total index, the DSM-IV Hyperactive-Impulsive index, and Conners’ ADHD index in quantile regression models at the 75th percentile. Associations of estimated infant serum PCB-153 levels with ADHD-related behavior were most suggestive for performance on the DSM-IV Hyperactive-Impulsive and Conners’ ADHD indices (Figure 3), but these associations, although statistically significant through age 6 months, diminished over time. Previous studies associating postnatal PCB exposures with adverse behavior have typically been done in animal models or human populations with higher PCB levels than were observed in the New Bedford Cohort. For example, monkeys exposed to a mixture of PCBs during their first 20 weeks of life (levels of 1,694–3,560 ng/g lipids) showed reduced performance on tasks of nonspatial discrimination, spatial delayed alternation, and fixed interval schedules at the age of 2.5–5 years, all of which are correlates of ADHD-related behaviors, including impulse control, in humans (Rice 2000). Similarly, our previous study of Inuit infants suggested that activity, in particular, may be susceptible to PCB exposure via breastfeeding in the first year of life but at higher exposure levels than were observed in New Bedford children (Verner et al. 2010). Specifically, in 150 Inuit infants from Northern Quebec (Canada), estimated postnatal serum PCB-153 levels during the 4th month of life (mean, 211 ng/g lipids; range, 5–1,548) were associated with increased spontaneous activity at 11 months (Verner et al. 2010). Plusquellec et al. (2010) observed an association between plasma PCB-153 levels at 5 years of age (mean, 153 ng/g lipids; range, 8–780) and increased concurrent spontaneous activity among 87 Inuit preschoolers. Although studies among Inuit children suggest that postnatal exposure to PCBs increases activity levels, Jacobson et al. (1990) reported an inverse association between total serum PCB levels at 4 years (mean serum total PCBs, 2.0–2.2 ng/mL) and a concurrent composite activity rating. Of note, no associations have been detected between attention and postnatal PCB exposure (Jacobson and Jacobson 2003; Plusquellec et al. 2010; Verner et al. 2010).

Certain factors such as genetics (Sharp et al. 2009) and early-life exposures (e.g., maternal smoking) (Latimer et al. 2012) are determinants of later-life inattentive and hyperactive-impulsive behaviors. Children who demonstrated ADHD-like behaviors at 8 years of age, that is, those at higher percentiles of CRS-T scores, were likely predisposed to these behaviors during development, when prenatal and lactational exposures to PCBs occurred. Interestingly, the association between cord serum PCB-153 and ADHD-related behaviors was stronger at the 75th percentile than at the 50th percentile of CRS-T scores. A possible interpretation of these findings is that predisposed individuals may be more vulnerable to PCB neurotoxic effects. Evidence of enhanced susceptibility to neurobehavioral toxicants among children who demonstrate suboptimal neurobehavioral performance has been reported by others. For example, in a study of childhood lead exposure and end-of-school-grade test scores, Miranda et al. (2009) observed stronger adverse associations between lead and test scores among children with worse compared with better scores. This hypothesis would need to be more thoroughly evaluated in prospective studies where more detailed information on predisposing factors, including genetic factors, is available.

Despite the fact that children were living near a PCB-contaminated site (Choi et al. 2006), PCB levels measured in cord serum were relatively low compared with other longitudinal cohort studies in North America and Europe (Longnecker et al. 2003). Low-level exposures experienced by the New Bedford Cohort children may explain the lack of clear association between postnatal PCB exposure and ADHD-related behaviors. In addition, limitations to our study may underlie our inability to detect a consistent association between estimated postnatal PCB levels and ADHD-related behavior. The pharmacokinetic model we used to estimate postnatal PCB levels, which considers exposure only through transplacental transfer and breastfeeding, was validated in cohorts where breastfeeding was highly prevalent and the resolution in the duration of breastfeeding was higher (continuous vs. categorical) (Verner et al. 2013). The low prevalence of breastfeeding (i.e., 54% of children were ever breastfed, 40% more than a month) and the limited resolution in the duration of breastfeeding measure, a model parameter that was highly sensitive in global sensitivity analyses of the pharmacokinetic model (Verner et al. 2013), likely introduced measurement error in the exposure predictions and consequently reduced our power to detect associations. Therefore, we cannot rule out the possibility that the absence of consistent associations of ADHD-related behaviors with postnatal PCBs is attributable to the reduced precision of postnatal exposure estimates in the New Bedford Cohort. Additional limitations to our study include pharmacokinetic model assumptions (e.g., postpartum weight changes, proportion of food intake attributable to breast milk during partial breastfeeding, constant maternal daily dose, postnatal exposure restricted to breastfeeding) and the lack of information on subject-specific cord blood lipids. Although these limitations may have diminished our ability to detect modest associations with postnatal exposure to PCBs, the use of a pharmacokinetic model enabled us to retrospectively investigate early PCB exposures in a cohort with no measured levels in infancy. Our results represent an important step toward assessing the potential for PCB exposures in infancy to affect subsequent behavioral development.

## Conclusions

Epidemiologic and experimental evidence suggests that hyperactive or impulsive behavior may be associated with postnatal PCB exposures (Holene et al. 1998; Rice 2000; Verner et al. 2010). To the best of our knowledge, this is the first study to assess the neurobehavioral impact of postnatal PCB exposures at low levels characteristic of contemporaneous general population samples. In the context of low-level exposure and limitations in postnatal exposure estimation where 60% of children were not breastfed or breastfed for < 1 month, our findings were inconclusive regarding the potential modest adverse impact of low-level PCB exposures in infancy on hyperactive or impulsive behavior in later childhood. Our results using quantile regression at the 75th percentile of CRS-T scores suggest that children observed to have more ADHD-like behavior at age 8 years, who were possibly predisposed by genetic and early-life environmental factors, may be more susceptible to both prenatal and postnatal exposure to PCBs. Further research is warranted to address the potential for low-level PCB exposure in infancy to affect later child neurobehavior and to assess sources of increased susceptibility to postnatal PCB effects.

## Supplemental Material

(314 KB) PDF
