# Supplementary material for: Measured Prenatal and Estimated Postnatal Levels of Polychlorinated Biphenyls (PCBs) and ADHD-Related Behaviors in 8-Year-Old Children
Source: Environ Health Perspect. 2015 Mar 13;123(9):888–94. doi: 10.1289/ehp.1408084 (PMC4559949; doi:10.1289/ehp.1408084)
Supplement: (314 KB) PDF [file ehp.1408084.s001.acco.pdf]

Environ Health Perspect
doi: 10.1289/ehp.1408084

Note to Readers: ***EHP*** **strives to ensure that all journal content is accessible to all readers. However, some figures and Supplemental Material published in** ***EHP*** **articles may not conform to 508 standards due to the complexity of the information being presented. If you need assistance accessing journal content, please contact ehp508@niehs.nih.gov. Our staff will work with you to assess and meet your accessibility needs within 3 working days.**

Supplemental Material

Measured Prenatal and Estimated Postnatal Levels of Polychlorinated Biphenyls (PCBs) and ADHD-Related Behaviors in 8-Year-Old Children

Marc-André Verner, Jaime E. Hart, Sharon K. Sagiv, David C. Bellinger, Larisa M. Altshul, and Susan A. Korrick

Table of contents

Figure S1. Distribution of serum lipid PCB-153 levels measured at birth and estimated thereafter through age 12 months for all children included in our analyses (n=441). This distribution includes data from breastfed and non-breastfed children. The y axis is in log scale to allow better representation of lower exposure percentiles.

Figure S2. Spearman rank correlation coefficients between estimated serum PCB-153 levels during infancy (1 to 12 months) and measured cord serum PCB-153 levels and total duration of breastfeeding.

Figure S3A. Sensitivity analyses. Quantile regression effect estimates for the 50th (top graphs) and 75th percentiles (bottom graphs) of scores for the Conners' Rating Scale for Teachers (CRS-T) ADHD-related indices (n=441). Circles represent the change in CRS-T score at age 8 for an interquartile range (IQR) increase in serum lipid PCB-153 levels measured in cord serum at birth and estimated for each month of infancy (time [months] and IQRs [ng/g lipids]: 0 (birth)=42; 1=49; 2=49; 3=44; 4=44; 5=45; 6=42; 7=42; 8=40; 9=39; 10=37; 11=36; 12=34). Error bars represent the 95% confidence intervals. Models were adjusted for maternal pre-pregnancy weight, gestational weight gain, characteristics at delivery (age, marital status, education, parity),

seafood consumption during pregnancy, use of tobacco and alcohol during pregnancy and use of illicit drugs in the year before delivery, and intellectual quotient (IQ) at 8-year follow-up; total household income (at delivery) and Home Observation for Measurement of the Environment (HOME) score at 8-year assessment; and child sex, race, cord blood lead level, ADHD medication use, school type and age at CRS teacher evaluation. Quantile regression models were also adjusted for total duration of breastfeeding.

Figure S3B. Sensitivity analyses. Quantile regression effect estimates for the 50th (top graphs) and 75th percentiles (bottom graphs) of scores for the Conners' Rating Scale for Teachers (CRS-T) ADHD-related indices (n=441). Circles represent the change in CRS-T score at age 8 for an interquartile range (IQR) increase in serum lipid PCB-153 levels estimated for each month of infancy (time [months] and IQRs [ng/g lipids]: 1=49; 2=49; 3=44; 4=44; 5=45; 6=42; 7=42; 8=40; 9=39; 10=37; 11=36; 12=34. Error bars represent the 95% confidence intervals. Models were adjusted for maternal pre-pregnancy weight, gestational weight gain, characteristics at delivery (age, marital status, education, parity), seafood consumption during pregnancy, use of tobacco and alcohol during pregnancy and use of illicit drugs in the year before delivery, and intellectual quotient (IQ) at 8-year follow-up; total household income (at delivery) and Home Observation for Measurement of the Environment (HOME) score at 8-year assessment; and child sex, race, cord blood lead level, ADHD medication use, school type and age at CRS teacher evaluation. Quantile regression models were also adjusted for measured cord serum PCB-153 levels.

Figure S3C. Sensitivity analyses. Quantile regression effect estimates for the 50th (top graphs) and 75th percentiles (bottom graphs) of scores for the Conners' Rating Scale for Teachers (CRS-T) ADHD-related indices in the subset of children who were breastfed (n=239). Circles represent the change in CRS-T score at age 8 for an interquartile range (IQR) increase in serum PCB-153 levels measured in cord serum at birth and estimated for each month of infancy (time [months] and IQR [ng/g lipids]: 0 (birth)=46; 1=66; 2=72; 3=62; 4=66; 5=75; 6=71; 7=68; 8=72; 9=76; 10=71; 12=69). Error bars represent the 95% confidence intervals. Models were adjusted for maternal pre-pregnancy weight, gestational weight gain, and characteristics at delivery (age, marital status, education, parity), seafood consumption during pregnancy, use of tobacco and alcohol during pregnancy and use of illicit drugs in the year before delivery, and intellectual quotient (IQ) at 8-year follow-up; total household income (at delivery) and Home Observation for Measurement of the Environment (HOME) score at 8-year assessment; and child sex, race, cord blood lead level, ADHD medication use, school type and age at CRS teacher evaluation.

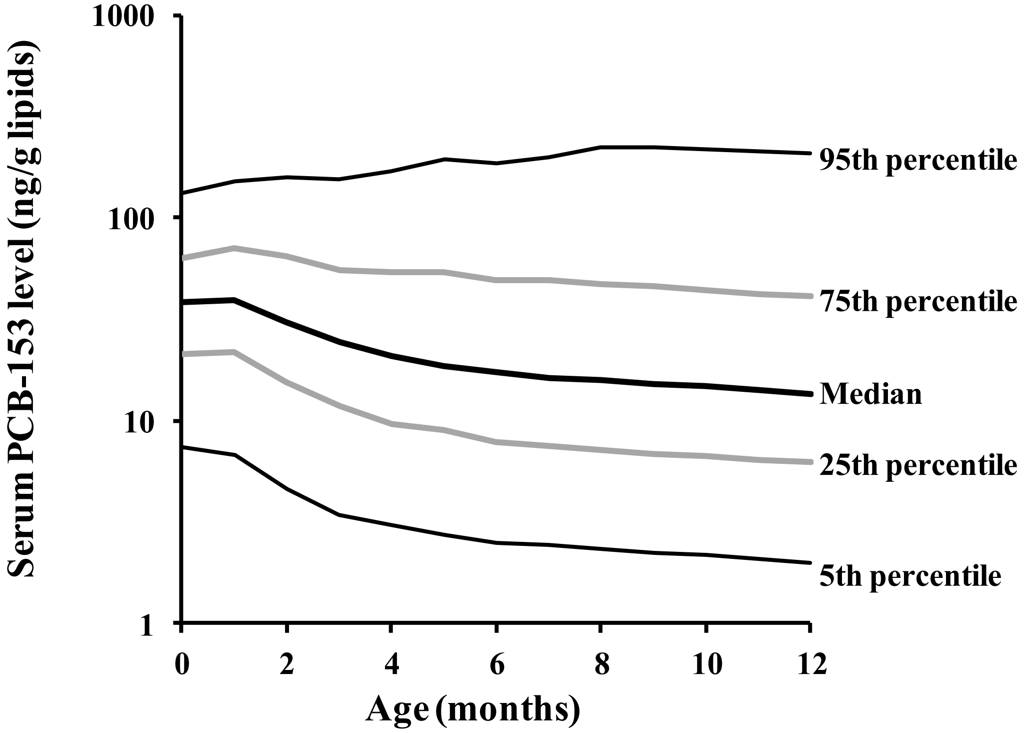

Figure S1. Distribution of serum lipid PCB-153 levels measured at birth and estimated thereafter through age 12 months for all children included in our analyses (n=441). This distribution includes data from breastfed and non-breastfed children. The y axis is in log scale to allow better representation of lower exposure percentiles.

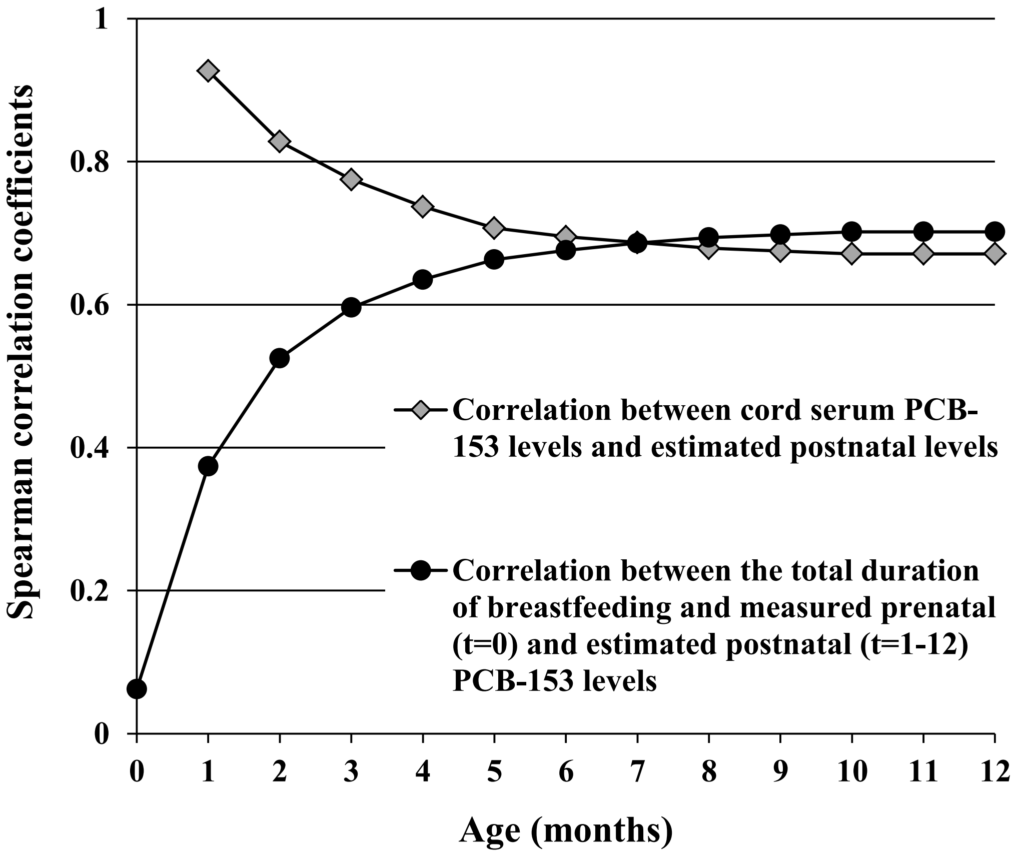

Figure S2. Spearman rank correlation coefficients between estimated serum PCB-153 levels during infancy (1 to 12 months) and measured cord serum PCB-153 levels and total duration of breastfeeding.

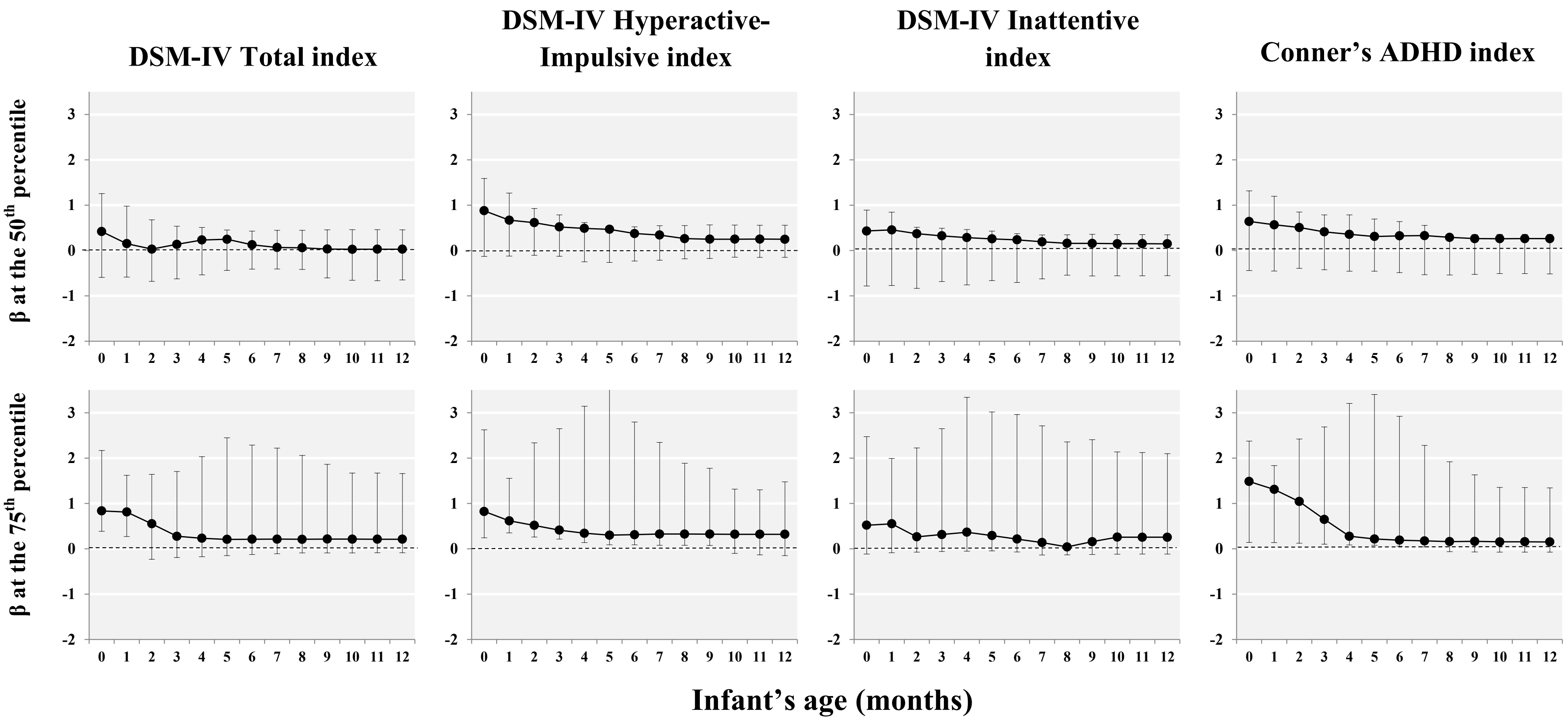

Figure S3A. Sensitivity analyses. Quantile regression effect estimates for the 50th (top graphs) and 75th percentiles (bottom graphs) of scores for the Conners' Rating Scale for Teachers (CRS-T) ADHD-related indices (n=441). Circles represent the change in CRS-T score at age 8 for an interquartile range (IQR) increase in serum lipid PCB-153 levels measured in cord serum at birth and estimated for each month of infancy (time [months] and IQRs [ng/g lipids]: 0 (birth)=42; 1=49; 2=49; 3=44; 4=44; 5=45; 6=42; 7=42; 8=40; 9=39; 10=37; 11=36; 12=34). Error bars represent the 95% confidence intervals. Models were adjusted for maternal pre-pregnancy weight, gestational weight gain, characteristics at delivery (age, marital status, education, parity), seafood consumption during pregnancy, use of tobacco and alcohol during pregnancy and use of illicit drugs in the year before delivery, and intellectual quotient (IQ) at 8-year follow-up; total household income (at delivery) and Home Observation for Measurement of the Environment (HOME) score at 8-year assessment; and child sex, race, cord blood lead level, ADHD medication use, school type and age at CRS teacher evaluation. Quantile regression models were also adjusted for total duration of breastfeeding.

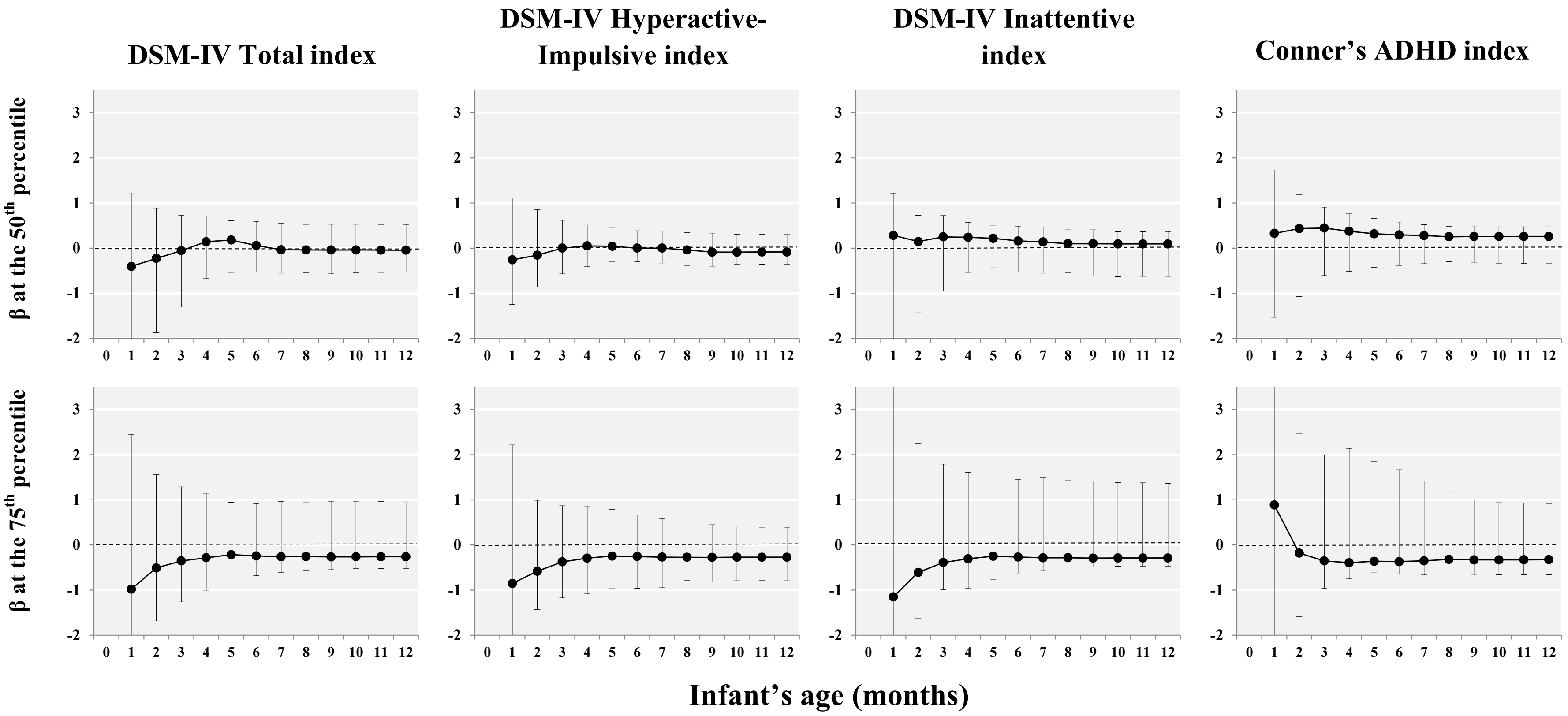

Figure S3B. Sensitivity analyses. Quantile regression effect estimates for the 50th (top graphs) and 75th percentiles (bottom graphs) of scores for the Conners' Rating Scale for Teachers (CRS-T) ADHD-related indices (n=441). Circles represent the change in CRS-T score at age 8 for an interquartile range (IQR) increase in serum lipid PCB-153 levels estimated for each month of infancy (time [months] and IQRs [ng/g lipids]: 1=49; 2=49; 3=44; 4=44; 5=45; 6=42; 7=42; 8=40; 9=39; 10=37; 11=36; 12=34. Error bars represent the 95% confidence intervals. Models were adjusted for maternal pre-pregnancy weight, gestational weight gain, characteristics at delivery (age, marital status, education, parity), seafood consumption during pregnancy, use of tobacco and alcohol during pregnancy and use of illicit drugs in the year before delivery, and intellectual quotient (IQ) at 8-year follow-up; total household income (at delivery) and Home Observation for Measurement of the Environment (HOME) score at 8-year assessment; and child sex, race, cord blood lead level, ADHD medication use, school type and age at CRS teacher evaluation. Quantile regression models were also adjusted for measured cord serum PCB-153 levels.

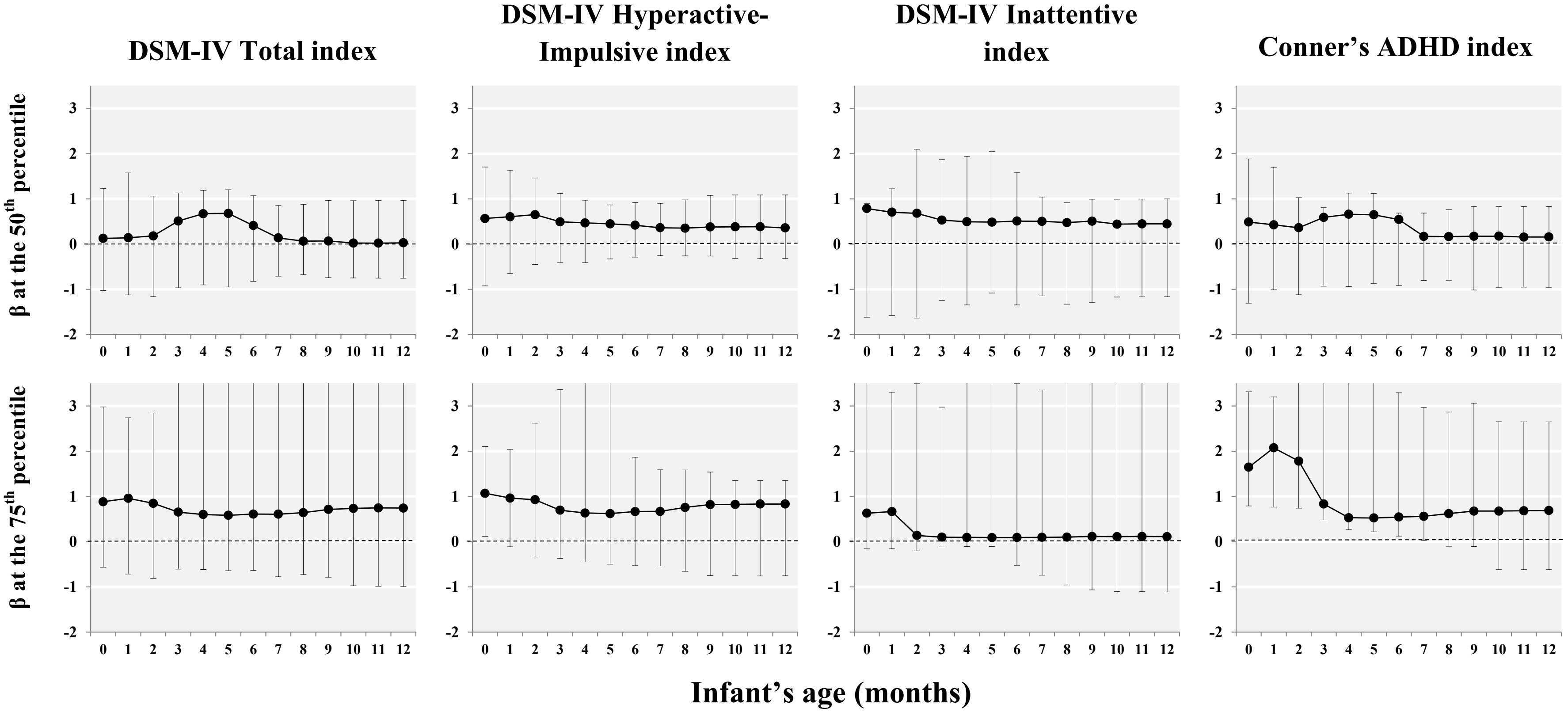

Figure S3C. Sensitivity analyses. Quantile regression effect estimates for the 50th (top graphs) and 75th percentiles (bottom graphs) of scores for the Conners' Rating Scale for Teachers (CRS-T) ADHD-related indices in the subset of children who were breastfed (n=239). Circles represent the change in CRS-T score at age 8 for an interquartile range (IQR) increase in serum PCB-153 levels measured in cord serum at birth and estimated for each month of infancy (time [months] and IQR [ng/g lipids]: 0 (birth)=46; 1=66; 2=72; 3=62; 4=66; 5=75; 6=71; 7=68; 8=72; 9=76; 10=71; 12=69). Error bars represent the 95% confidence intervals. Models were adjusted for maternal pre-pregnancy weight, gestational weight gain, and characteristics at delivery (age, marital status, education, parity), seafood consumption during pregnancy, use of tobacco and alcohol during pregnancy and use of illicit drugs in the year before delivery, and intellectual quotient (IQ) at 8-year follow-up; total household income (at delivery) and Home Observation for Measurement of the Environment (HOME) score at 8-year assessment; and child sex, race, cord blood lead level, ADHD medication use, school type and age at CRS teacher evaluation.
